# The genetic basis of incipient sexual isolation in *Drosophila melanogaster*

**DOI:** 10.1098/rspb.2024.0672

**Published:** 2024-07-24

**Authors:** Akihiko Yamamoto, Wen Huang, Mary Anna Carbone, Robert R. H. Anholt, Trudy F. C. Mackay

**Affiliations:** ^1^ Program in Genetics, W. M. Keck Center for Behavioral Biology and Department of Biological Sciences, North Carolina State University, Raleigh NC, Raleigh, NC 27695-7614, USA; ^2^ Department of Entomology and Plant Pathology, North Carolina State University, Raleigh, NC, USA; ^3^ Department of Animal Science, Michigan State University, 474 S Shaw Lane, East Lansing, MI, USA; ^4^ Center for Fungal Research and Department of Plant and Microbial Biology, North Carolina State University, Raleigh, NC, USA; ^5^ Center for Human Genetics and Department of Genetics and Biochemistry, Clemson University, 114 Gregor Mendel Circle, Greenwood, SC, USA

**Keywords:** speciation, genome-wide association study, mating behaviour, Drosophila genetic reference panel

## Abstract

Speciation is a fundamental evolutionary process but the genetic changes accompanying speciation are difficult to determine since true species do not produce viable and fertile offspring. Partially reproductively isolated incipient species are useful for assessing genetic changes that occur prior to speciation. *Drosophila melanogaster* from Zimbabwe, Africa are partially sexually isolated from other *D. melanogaster* populations whose males have poor mating success with Zimbabwe females. We used the North American *D. melanogaster* Genetic Reference Panel (DGRP) to show that there is significant genetic variation in mating success of DGRP males with Zimbabwe females, to map genetic variants and genes associated with variation in mating success and to determine whether mating success to Zimbabwe females is associated with other quantitative traits previously measured in the DGRP. Incipient sexual isolation is highly polygenic and associated with the common African inversion *In(3R)K* and the amount of the sex pheromone 5,9-heptacosadiene in DGRP females. We functionally validated the effect of eight candidate genes using RNA interference to provide testable hypotheses for future studies investigating the molecular genetic basis of incipient sexual isolation in *D. melanogaster*.

## 1. Introduction

The genetic basis of speciation—the genetic changes causing the splitting of a panmictic population into two reproductively isolated species—is difficult to determine, since, by definition, complete reproductive isolation is refractory to genetic mapping. Many insights have been gained by mapping the genetic basis of divergence between closely related species with incomplete reproductive isolation, at least in laboratory settings [1]. However, this genetic differentiation includes changes that occurred following speciation as well as those causing or accompanying speciation.

Populations of *Drosophila melanogaster* from Zimbabwe (Z), Africa are genetically differentiated from cosmopolitan (C) populations worldwide [2], and there is partial sexual isolation between Z and C populations [3–5]. This presents an ideal scenario to investigate the early stages of sexual isolation—thought to be the first stage in speciation [6]—in a genetically tractable model system. The partial sexual isolation between Z and C populations is asymmetric and is driven by female choice: Z females do not mate with C males, but all other combinations of mating pairs are successful [3–5]. Chromosome substitution analyses indicated that genes contributing to the asymmetric sexual isolation were autosomal, with a larger contribution from the third than the second chromosome [3–5]. Mapping factors on the third chromosome by linkage to visible markers identified three regions associated with Z female mating preference [7]. However, further high-resolution mapping was stymied by the large number of segregating inversions between Z and C populations [8].

Two strategies have been used to gain insight into the genetic differences associated with the partial sexual isolation between Z and C populations. One is association mapping using variants in candidate genes that are divergent between the two populations, and the other is searching for traits that are genetically correlated with the difference in mating behaviour between Z and C populations. These strategies were initially applied to differences in cuticular hydrocarbon (CHC) profiles between the two populations [9–11], since CHCs are used as mating cues in both sexes and are divergent between Z and C populations. However, these studies were limited by using small numbers of Z and C strains.

Here, we extend the genetic and trait association strategies to the 205 inbred, sequenced C lines of the *D. melanogaster* Genetic Reference Population (DGRP) [12,13] and an outbred advanced intercross population (AIP) derived from a subset of DGRP lines. We found significant genetic variation for the mating behaviour of Z females with the DGRP (C) males and performed genome-wide association (GWA) mapping of variants associated with DGRP male mating ability with Z females. Since the DGRP has been assessed for over 100 quantitative traits, including CHC composition and ecologically relevant traits [14], we could also assess correlations of male DGRP traits with Z female mating behaviour. We used the AIP population to select for C males with increased mating to Z females and performed whole-genome sequencing to identify alleles associated with increased Z female preference. We then used RNA interference (RNAi) to functionally assess the genetic associations at the level of candidate genes. We found that the genetic basis of incipient sexual isolation between DGRP males and Z females is highly polygenic and associated with the presence of the common African polymorphic inversion *In(3R)K* as well as the amount of 5,9-heptacosadiene produced by DGRP females. We identified many candidate genes and variants in the DGRP associated with mating behaviour with Z females that can be used in future studies of the molecular genetic basis of incipient sexual isolation in *D. melanogaster*.

## 2. Material and methods

### (a) *Drosophila* stocks

The 205 inbred, sequenced DGRP lines were derived from inseminated females collected in Raleigh, NC, USA [12,13]. The Z30 strain from Zimbabwe was a gift from Dr C. F. Aquadro, Cornell University. Oregon and Samarkand are common C wild type stocks, unrelated to the DGRP lines. RNAi lines were purchased from the Vienna *Drosophila* Resource Center. The *GAL4* driver lines *Act-GAL4* (*P{Act5C-GAL4}25FO1*) and *Ubi-GAL4* (*P{Ubi-GAL4}2*) were obtained from the Bloomington *Drosophila* Stock Center and their major chromosomes that do not contain the drivers were replaced with *Canton-S-B* chromosomes [15] (*CSB, w* ^1118^, a C strain) to minimize background genotype effects. A new driver stock, *Ubi-GAL4[156]*, was created by introducing the original *Ubi-GAL4* transgene onto the third chromosome of *CSB* by *Δ2–3* transposase-mediated hopping.

The AIP was constructed from 40 DGRP lines: DGRP_208, DGRP_301, DGRP_303, DGRP_304, DGRP_306, DGRP_307, DGRP_313, DGRP_315, DGRP_324, DGRP_335, DGRP_357, DGRP_358, DGRP_360, DGRP_362, DGRP_365, DGRP_375, DGRP_379, DGRP_380, DGRP_391, DGRP_399, DGRP_427, DGRP_437, DGRP_486, DGRP_514, DGRP_517, DGRP_555, DGRP_639, DGRP_705, DGRP_707, DGRP_712, DGRP_714, DGRP_730, DGRP_732, DGRP_765, DGRP_774, DGRP_786, DGRP_799, DGRP_820, DGRP_852, DGRP_859. These lines were crossed in a round-robin mating design in generation 1 (i.e. line 1 females by line 2 males, line 2 females by line 3 males, …, line 40 females by line 1 males) to create 40 F1 genotypes. In the second generation, we performed another round-robin cross between pairs of F1 genotypes (i.e. line 1/line 2 F1 females by line 3/line 4 F1 males) to create a highly heterozygous population. At generation 3, 10 replicate populations were established, each with one female and one male from each of the 40 generation 2 crosses, and flies were allowed to lay eggs for 2 days to minimize natural selection via larval competition. The AIP was maintained from generation 4 in 10 bottles with four females and four males from each of the 10 bottles of the previous generation, for a census population size of 800. All stocks were raised on standard cornmeal/molasses/agar medium at 25°C.

### (b) Mating assay

Five virgin Z30 females and 10 C males were placed without anaesthesia in a vial (25 mm diameter × 95 mm high) with 1 ml of medium. All flies were between 4 and 7 days old. Mating was observed directly and the time to copulation recorded. Each copulating couple was immediately removed using a mouth aspirator through a slit window in a sponge plug [16]. Mating was observed for 30 min, 1 h and/or 2 h, depending on the experiment. All assays were conducted between 8.00 and 11.00 under full lighting at 25°C.

### (c) Quantitative genetics of male mating success with Z30 females in the DGRP

We partitioned the phenotypic variance in male mating success with Z30 females by a mixed model in which the response variable was the mating success rate and the independent variable was the genotype of the flies as a random effect. Broad sense heritability (*H*2) is estimated as $H^{2}=\frac{\sigma_{g}^{2}}{\sigma_{g}^{2}+\sigma_{e}^{2}}$ , where $\sigma_{g}^{2}$ is the variance component owing to genotype and $\sigma_{e}^{2}$ is the variance component owing to random environmental effect. Correlations between male mating success and other quantitative traits were computed as Pearson’s correlation coefficient of line means between two traits.

### (d) Genome-wide association (GWA) analysis in the DGRP

We performed a GWA analysis [13] for mating success of DGRP males with Z30 females using the 2 525 695 SNPs and indels with minor allele frequencies greater than 0.02. Briefly, raw sequence reads from each DGRP line were aligned to the reference sequence and then joint genotyping for inbred lines [17] was used to perform integrated genotyping. The 2 525 695 SNPs and indels with minor allele frequencies greater than 0.02 were used in the GWA analysis. To perform the GWA analysis, raw data phenotypic data were adjusted for the effects of *Wolbachia* infection and major polymorphic inversions ([*In(2L)t*, *In(2R)NS*, *In(3R)P*, *In(3R)K* and *In(3R)Mo*] and a linear model (*y =* **X** *b + Zu+e*, where *y* is the adjusted phenotypic values, **X** is the design matrix for the fixed SNP effect *b*, **Z** is the incidence matrix for the random polygenic effect *u* and *e* is the residual) was fit to the adjusted line means. The linear model was fit using FastLMM v. 1.09 [18]. This analysis accounts for effects of Wolbachia infection, cryptic relatedness owing to major inversions, and residual polygenic relatedness. In addition, we performed a similar GWA analysis without correcting for effects of inversions to identify variants within inversions that may contribute to phenotypic variation.

### (e) xQTL mapping

We performed xQTL mapping following a similar procedure as previously described [13]. A total of 500 male flies at generation 156 were assessed for their mating with Z30 females and 50 fastest maters were selected to form a high mating pool and 50 randomly selected flies were selected to form a control pool. Four biological replicates were performed for each of the high mating and control pools. The pooled flies were sequenced, and the sequences were analysed following a previously described approach [19] (https://github.com/qgg-lab/xqtl). Briefly, reads were mapped to the reference genome and counts of alleles were computed to compare allele frequencies of the high mating and control pool. We used a *Z* score test in the form of $Z=\frac{\bar{p_{2}}-\bar{p_{1}}}{\sqrt{\sum w_{2}p_{2}\left(1-p_{2}\right)\left(\frac{1}{2n_{2}}+\frac{1}{c_{2}}\right)+\;\sum w_{1}p_{1}\left(1-p_{1}\right)\left(\frac{1}{2n_{1}}+\frac{1}{c_{1}}\right)}}$ , where $\bar{p_{2}}$ and $\bar{p_{1}}$ are average allele frequencies in the two groups (high mating and control) across replicates, *n_1_* and *n_2_* are numbers of flies in the pools, and *c_1_* and *c_2_* are the sequencing coverage in the pools. The variance of the allele frequency was averaged with weights (*w_2_*, *w_1_*) according to sequencing depths. *p*-values were obtained by comparing the *Z* score to standard normal distribution.

### (f) Selection experiments

At AIP generation 30, two replicates of 300 males each were assessed for mating during 1 h with Z30 females. Groups of 10 AIP males and five Z30 females were placed in vials, and the first 40 males to mate with Z30 females in each replicate were selected and mated with 40 virgin AIP siblings. Selection was continued for 18 generations. Initially, a single control population was established by observing the mating of 300 AIP males with Z30 females, and randomly selecting 40 males to be parents of the next generation by crossing with 40 AIP females. A second control population was established from the first at generation 5; both were then maintained by scoring mating of 150 males and randomly selecting 40 males to cross with 40 AIP females. At selection generation 18, the copulation latency of males from the selection and control lines was assessed using F1 hybrid females from a cross between the C strains Oregon and Samarkand. Also at selection generation 18, we paired 600 males from each selection line with 300 Z30 females (60 vials) and collected the first 100 males to mate from each selection line and froze them at −80°C for subsequent DNA sequencing. We also collected and froze 100 randomly selected males from the two generation 18 control populations for subsequent DNA sequencing. The comparison of allele frequencies was done as described above for the xQTL mapping except that the control population and the selected population were not paired, we therefore performed comparisons for all possible pairs and stringently required that the minimal difference was above the threshold.

### (g) DNA sequencing

We sequenced one sample each containing pools of 100 males from the two replicate control and selection lines at selection generation 18 and four samples each containing pools of 50 males from each of the high and control single generation selection experiments. We homogenized the flies from each sample in Gentra Puregene Cell Lysis Solution (Qiagen) with ceramic beads using the TissueLyser (Qiagen). Genomic DNA was extracted using the Gentra Puregene Tissue Kit (Qiagen) and further purified with AMPure XP magnetic beads (Beckman Coulter). Genomic DNA was fragmented to 300–400 bp using ultrasonication (Covaris S220). Fragmented DNA was used to produce barcoded DNA libraries using NEXTflex DNA Barcodes (Bioo Scientific) with either the TruSeq DNA Library Prep Kit (Illumina) (selection Generation 18 samples) or an Illumina TruSeq compatible protocol (single generation selection samples). Libraries were quantified using Qubit dsDNA HS Kits (Life Technologies) and Bioanalyzer (Agilent Technologies) to calculate molarity. Libraries were then diluted to equal molarity and re-quantified. The four selection generation 18 samples were pooled together; and the eight single generation selection samples were pooled together. Pooled library samples were quantified again to calculate final molarity and then denatured and diluted to 14 pM. Pooled library samples were clustered on an Illumina cBot. The selection generation 18 samples were sequenced on one HiSeq2000 lane using 100 bp paired-end v3 chemistry, and the eight single generation selection samples were each sequenced on two Hiseq2500 high throughput lanes using 125 bp paired-end v4 chemistry.

### (h) RNA interference (RNAi)

We used three ubiquitously expressed *GAL4* drivers to knock down expression of selected candidate genes: *Act-GAL4*, *Ubi-GAL4* and *Ubi-GAL4 [156]*. All drivers are in the *CSB w* ^1118^ genetic background; *Act-GAL4* and *Ubi-GAL4* are maintained over a *CyO* balancer chromosome. We performed luciferase assays to assess the strengths of knock down for each *GAL4* driver. We crossed each driver to a *UAS*-Luciferase stock and collected *GAL4/UAS**-*** Luciferase F1 progeny as well as *CyO*/*UAS**-*** Luciferase F1 (control) progeny for *Act-GAL4* and *Ubi-GAL4*. The controls for *Ubi-GAL4 [156]* are the CSB/*UAS**-*** Luciferase F1 progeny from crossing CSB with the *UAS*-Luciferase stock. We prepared triplicate tissue homogenates from 10 F1 progeny from each cross using the Luciferase Cell Culture Lysis 5X Reagent (Promega) to extract total proteins by following the quick-freeze homogenization method outlined by the manufacturer. We quantified the resulting supernatants for their protein concentrations on a SpectraMax M2 (Molecular Devices) using the DC Protein Assay Kit II (BioRad). Luciferase activities were measured on a GloMax Luminometer (Promega) using the Steady-Glo Luciferase Assay System (Promega).

We selected 17 candidate genes to evaluate whether RNAi knockdown of gene expression affected mating performance with Z30 females based on several criteria: low *p*-value of association in any GWA analyses; gene overlap in more than one GWA analysis, functional annotations of candidate genes and availability of RNAi reagents. We crossed females of each driver line to the RNAi line and the appropriate co-isogenic control line and assessed mating of the F1 males from these crosses with Z30 females at 30 min, 1 h and 2 h, using 10 replicate vials each with 10 *UAS*-RNAi/*GAL4* males and five virgin Z30 females and 20 replicate vials each with 10 control/*GAL4* males and five virgin Z30 females. The exceptions were crosses for *Or67d* RNAi lines, in which the RNAi genotypes were used as male parents and there were 14 replicate vials with 10 males and five virgin Z30 females for each of the RNAi and control genotypes. Mating data were analysed using Fisher Exact tests of mating data for each RNAi line and appropriate control.

## 3. Results

### (a) Variation in DGRP male mating success with Z30 females

**Figure 1.**
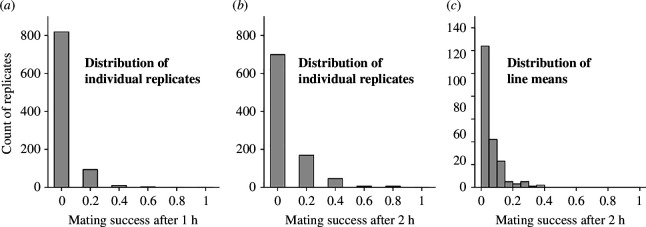
Distribution of DGRP male mating success with Z30 females. (*a*) Distribution of replicate level phenotype for mating success measured after 1 h. (*b*) Distribution of replicate level phenotype for mating success measured after 2 h. (*c*) Distribution of line means for mating success measured after 2 h.

We assessed whether the DGRP, a cosmopolitan (C) population, harboured genetic variation in male mating success with Z30 females, which showed a strong preference for Z30 males and avoided mating with C males [3–5]. We quantified mating success as the proportion of females that copulated in 1 or 2 h in a no-choice assay in vials with five Z30 females and 10 DGRP males. The mean proportion of successful matings within each line varied between 0 and 0.25 after 1 h, and 0 and 0.39 after 2 h, with respective means of 0.02 and 0.05 (figure 1; electronic supplementary material, table S1). There is substantial genetic variation among DGRP males that affect how Z30 females choose their mates. The broad sense heritability ( $H^{2}$ ) of ‘acceptability’ of DGRP males to Z30 females was $H^{2}$ = 0.27 (*p* = 1.98 × 10^−42^) for the 2 h time point, which was used for all subsequent analyses. The significant genetic variability in acceptability of DGRP males allows us to dissect factors contributing to such variation in the fully sequenced and deeply phenotyped DGRP.

The most abundant CHC moieties in *D. melanogaster* are also sex pheromones that affect mating behaviour [20], and female [9,10] and male [11] CHC composition have been implicated in the mating success of Z30 females. We have previously measured variation in CHC profiles in the DGRP lines [21] and can thus test these associations in a population with natural variation in CHC abundance. However, we did not find any significant associations of male hydrocarbons, including 7-tricosene and the relative proportion of 7-tricosene (electronic supplementary material, table S2A), in contrast to a previous report [11]. *Desat1* is involved in both the emission and perception of sex pheromones [22,23]. *Desat1* expression is genetically variable in the DGRP, with a broad sense heritability of *H* ^2^ = 0.69 in males [24]. However, variation in male *Desat1* expression is not associated with variation in DGRP male mating success with Z30 females (electronic supplementary material, table S2B). The failure to replicate the earlier associations with the relative amounts of male hydrocarbons and *Desat1* expression may be attributable to the larger number of C lines tested in this report.

Many other quantitative traits that could plausibly be genetically correlated with male mating success with Z30 females are genetically variable in the DGRP and have been measured under the same conditions as this study. We assessed the correlations of male aggressive behaviour [25], startle response, starvation resistance and chill coma recovery time [12], phototaxis [26], sleep traits and waking activity [27], DGRP male mating success with C females [28], body weight and body size [24], food consumption [29] and metabolic traits [24] (electronic supplementary material, table S2C–K). The only quantitative trait that is significantly, albeit moderately, correlated with DGRP male mating success with Z30 females is DGRP male mating success with Oregon/Samarkand F1 hybrid C females (*r* = 0.256, *p* = 0.00021; electronic supplementary material, table S2I), suggesting that the male component of the mating success trait is partially independent of the female genotype [28]. We compared the GWA results from DGRP male mating success to O/S hybrid females [28] with those from the GWA analyses for mating success of the same DGRP male genotypes with Z30 females (electronic supplementary material, table S3). Consistent with the inference that male mating success is partially independent of the female genotype and partially specific for Z30 females, only two genes were in common between the two analyses: *CG9850* and *CR32773*.

### (b) GWA analyses of male DGRP mating success with Z30 females

**Figure 2.**
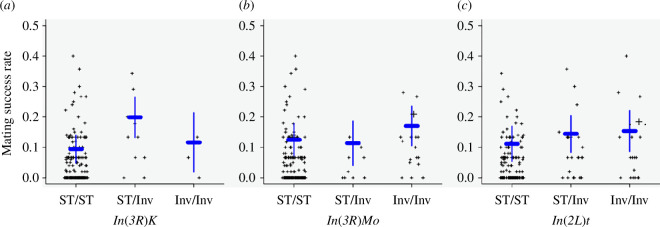
Effects of inversions on DGRP male mating success with Z30 females. (*a*) Effect of *In(3R)K* on mating success (*p* = 9.16 × 10^−5^, electronic supplementary material, table S3A). (*b*) Effect of *In(3R)Mo* on mating success (*p* = 4.58 × 10^−2^, electronic supplementary material, table S3A). (*c*) Effect of *In(2L)t* on mating success (*p* = 2.23 × 10^−2^, electronic supplementary material, table S3A). The blue horizontal bar represents least squares mean of each group and vertical bar represents 95% confidence interval.

*Drosophila melanogaster* populations are polymorphic for many chromosome inversions that often have population specific frequencies between African and C populations [30]. Standard and inverted sequences are genetically divergent owing to lack of recombination between them [12,30]. Therefore, we evaluated whether inversions segregating in the DGRP were associated with DGRP male mating success with Z30 females. We found that *In(3R)K* (proximal and distal breakpoints *3R*_7576289 and *3R*_21966092, respectively), which has a high frequency in African populations but is rare in C populations [30], has a large effect on DGRP mating success with C females (*p* = 9.16 × 10^−5^, electronic supplementary material, table S3A, figure 2*a*). The *In(3R)K*/ST inversion heterozygotes have the highest proportion of Z30 female matings relative to the standard karyotype (*p* = 1.77 × 10^−5^). In addition, *In(3R)Mo* (proximal and distal breakpoints *3R*_17232639 and *3R*_24857019, respectively), which is absent in Africa and rare in most C populations [30], but which has a fairly high frequency in the DGRP [12], is also associated with DGRP male mating success with Z30 females (*p* = 0.04, electronic supplementary material, table S3A, figure 2*b*). In this case, it is the homozygous inversion genotype that has the highest proportion of Z30 female matings relative to the standard karyotype (*p* = 1.61 × 10^−2^). Furthermore, *In(2L)t* (proximal and distal break points *2L*_13154180 and *2L*_2225744, respectively), which is common in African and C populations [30], is also associated with male mating success (*p* = 0.02; electronic supplementary material, table S3A, figure 2*c*) where the homozygous inversion genotype is associated with higher mating success (*p* = 0.02, electronic supplementary material, table S3A). These results indicated that the inversions may themselves contain variants that contribute to male mating success. Therefore, we performed two GWA analyses for variants at MAF > 0.02, one accounting for the effect of inversions on the trait [13], and one without using inversion status as covariates.

The top variants in the GWA analysis (reporting *p*‐value < 10^−5^) for which the effects of inversions were accounted for identified 156 variants in or near 115 genes (electronic supplementary material, table S3B and S3D). Four intronic variants in three genes (*mew*, *CG40470*, *wry*) were significant after applying a stringent Bonferroni correction for multiple tests (0.05/2 525 695 variants = 1.98 × 10^−8^). The top variants in the GWA analysis for which the effects of inversions were not used as covariates identified 266 variants in or near 182 genes (electronic supplementary material, table S3C and S3D). Six variants in five genes (*mew*, *CG40470*, *CG10226*, *CR44546*, *Or67d*) were significant after applying the Bonferroni correction. A total of 73 genes overlapped between the two GWA analyses, 42 were unique to the analysis corrected for inversions and 109 were unique to the analysis not corrected for inversions (electronic supplementary material, table S3D). The 224 genes identified in these analyses were enriched [31] for Gene Ontology terms involved in cellular signalling, including synaptic signalling and G-protein coupled receptor signalling, and several canonical signalling pathways (Decapentaplegic, Screw, Transforming growth factor beta) (electronic supplementary material, table S3E).

### (c) Extreme QTL (xQTL) mapping for male mating success with Z30 females

A complementary approach to GWA analysis using the DGRP lines is to use extreme QTL (xQTL) mapping [32] by selecting males from an outbred population that readily mate with Z30 females and comparing their allele frequencies genome-wide with equal numbers of randomly selected males. We constructed a highly heterozygous outbred AIP from a subset of 40 DGRP lines. We sequenced genomic DNA from four pools of 50 males each from the same AIP population at generation 156 that mated rapidly with Z30 females, and four pools of 50 randomly selected males from the same population. We identified 45 variants in or near 44 genes (*p* < 10^−5^) in this analysis (electronic supplementary material, table S4A and S4B). Only one gene (*CG42368*) was shared between the xQTL analysis and GWA analysis in the DGRP (electronic supplementary material, table S4C), which may be owing to context-dependent genetic effects [19].

### (d) Selection from an outbred population for increased Z30 female mating success

Heritable traits are expected to respond to directional artificial selection, during which genetic differentiation is expected to occur. We therefore performed a multi-generation selection experiment and sequenced selected and control populations to identify genomic regions that responded to selection. Unlike xQTL mapping, selection and drift both extend linkage disequilibrium (LD) in selection lines, reducing the resolution of mapping.

**Figure 3.**
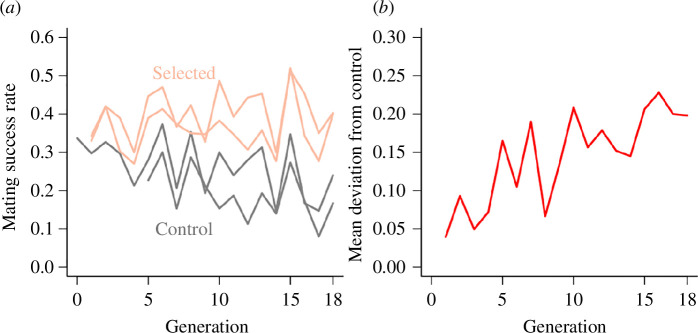
Response to 18 generations of selection from an AIP derived from 40 DGRP lines for male mating success with Z30 females. (*a*) Phenotypic trends in individual replicate populations. (*b*) Average effect of selection. The regression coefficients (*b*) of the deviations of selection lines from controls and respective *p*-values are *b* = 0.0080 (*p* = 4.31 × 10^−4^) for Replicate 1, *b* = 0.0096 (*p* = 6.38 × 10^−4^) for Replicate 2, and *b* = 0.0088 (*p* = 9.72 × 10^−5^) for the replicate averages (electronic supplementary material, table S5A).

We performed 18 generations of selection of C males for mating success to Z30 females from an outbred AIP. The selected lines reached more than 40% mating success in 1 h compared to the control lines by generation 18, which had an average mating success of 20% (figure 3; electronic supplementary material, table S5A). While there was considerable fluctuation, the mean deviation of selected lines from contemporary control lines showed a clear directional trend towards higher male mating success with Z30 females. Remarkably, the selection response remained even when mating to females from a tester C line. At generation 18, the average proportion of Oregon/Samarkand F1 hybrid females mating with control males was 0.567, while with selected males it was 0.729 (electronic supplementary material, table S5A). This result is consistent with the genetic correlation between mating success with Z30 females and that with Oregon/Samarkand F1 females (electronic supplementary material, table S2I). However, this correlation, although significant, was not large, suggesting that some component of male mating success (we do not know what this is) is partially independent of the female genotype while other components are specific for Z30 females. We compared the GWA results from DGRP male mating success to O/S hybrid females with those from the GWAS for mating success of the same DGRP male genotypes with Z30 females. Consistent with the inference that male mating success is partially independent of the female genotype and partially specific for Z30 females, only two genes were in common between the two analyses: *CG9850* and *CR32773*.

To identify genomic regions that responded to selection, we sequenced genomic DNA from pools of 100 males of the replicate control and selected lines. We identified 2223 variants in or near 968 genes with divergent allele frequencies between the selected and control lines at *p* < 10^−5^ (electronic supplementary material, table S5B and S5C). The homozygous DGRP, xQTL mapping in the outbred population and multi-generation selection experiment are complementary and have different strengths and weaknesses. A total of 31 genes were in common between two of these analyses: 23 between the DGRP and multi-generation selection analyses, one between the DGRP and xQTL mapping analyses and seven between the multi-generation selection and xQTL mapping analyses (electronic supplementary material, table S4C).

### (e) RNAi of candidate genes

To functionally validate candidate genes identified in these experiments, we used RNAi lines to specifically knockdown expression of candidate genes. We first assessed the strength of three ubiquitously expressed *GAL4* drivers (*Act-GAL4*, *Ubi-GAL4* and *Ubi-GAL4 [156]*) using a luciferase assay. Based on this assay, the relative strengths of the *GAL4* drivers are *Act-GAL4* > *Ubi* GAL4 > *Ubi-GAL4[156]* (electronic supplementary material, table S6A). We chose 17 candidate genes from the DGRP and selection analyses for functional analyses. We included genes that had a small *p*-value from any analysis (*CG33144*, *CG42458*, *CG44837*, *frac*, *mew*, *wry*); were present in two analyses (*btsz*, *CG34114*, *nmo*, *Rbp6*, *tkv*); and had involvement in sensory perception (*dpr1*, *Or67d*), nervous system development and function (*C15*) and/or brain gene expression (*CG1136*, *CG42672*, *jvl*). We evaluated their effects on mating with Z30 females using the three ubiquitously expressed *GAL4* drivers (electronic supplementary material, table S6B). RNAi of eight of these genes (47.1%) affected mating success of males with Z30 females. RNAi of *CG44837* increased male mating success with Z30 females compared to the control; and RNAi of *btsz*, *C15*, *CG1136*, *CG42672*, *dpr1*, *nmo* and *jvl* decreased male mating success with Z30 females relative to the control (electronic supplementary material, table S6B).

### (f) Female pheromones

Most previous studies of the genetic basis of the differences in mating behaviour of Z and C populations have focused on CHCs that are sex pheromones. The most common female CHC in African and Caribbean populations is 5,9-heptacosadiene, while the most common female CHC in cosmopolitan populations is 7,11-heptacosadiene. The abundance of 5,9-heptacosadiene has been associated with a regulatory polymorphism in *Desat2*, which has a 16 bp deletion in C populations and an intact allele in Z populations [9,10]. Interestingly, there is significant variation in the amount of 5,9-heptacosadiene among DGRP females [21], with a broad sense heritability of $H^{2}$ = 0.93. This might be partially attributable to the presence of the African *Desat2* allele in 17 DGRP lines. However, this allele is not perfectly associated with either variation in the amount of 5,9-heptacosadiene [21] or mating success with Z30 females (electronic supplementary material, table S7A). For example, DGRP_105 and DGRP_235 both have the African *Desat2* allele; but DGRP_105 has low amounts of 5,9-heptacosdiene and a low proportion of mating with Z30 females; while DGRP_235 has high amounts of 5,9-heptacosdiene and no mating with Z30 females. The correlation between amount of 5,9-heptacosdiene and Z30 mating success in the 17 DGRP lines with the African *Desat2* allele is *r* = 0.077 (*p* = 0.769) (electronic supplementary material, table S7A). Furthermore, *Desat2* is not expressed in any of the DGRP lines, including those with the African *Desat2* allele [24]. We performed a GWA analysis of the amount of 5,9-heptacosadiene in DGRP females (electronic supplementary material, table S7) using the data of Dembeck *et al.* [21]. The amount of 5,9-heptacosadiene is associated with *In(3R)K* inversion status for both homozygous and heterozygous inversions (electronic supplementary material, table S7B), and with a total of 612 variants (electronic supplementary material, table S7C) in or near 341 genes (electronic supplementary material, table S7D). The African *Desat2* allele was not among the top-associated variants. However, 17 genes overlapped between the GWA analyses for Z30 female mating success and amount of 5,9-heptacosadiene in DGRP females (electronic supplementary material, table S7D), of which three genes (*btsz*, *CG18208* and *Octbeta2R*) are located in *In(3R)K* and are good candidates for the strong association of this inversion with both Z30 mating success and amount of 5,9-heptacosadiene in DGRP females.

## 4. Discussion

We performed three unbiased GWA analysis screens to detect DGRP genes and variants associated with mating success with Z30 females that have different advantages and disadvantages. The DGRP GWA analysis is adequately powered to detect common variants with fairly large effects [14], but not rare variants; and the DGRP has high mapping precision because LD declines rapidly with physical distance in this population [12,13]. The analysis of allele frequency divergence from multiple generations of selection has the power to detect rare variants that increase in frequency in the selection lines, but little power to disentangle the effects of selection and genetic drift for common alleles with only two replicate selection and control lines; further, selection and drift both cause LD, reducing the precision of mapping [33]. The single generation selection experiment can detect common alleles associated with Z30 female mating because the effect of drift and LD is less than the multi-generation selection experiment owing to the large effective population size of the AIP, but it has little power to detect rare alleles. Both AIP designs have reduced genetic variation compared to the entire DGRP. Finally, the DGRP lines are inbred while the AIP population is outbred, and there is inbreeding depression for male mating behaviour. The mean mating frequency to Z30 females of the DGRP lines used as parents for the AIP is 0.06 (electronic supplementary material, table S2), while the mean mating frequency to Z30 females of AIP males at generation 0 of the selection experiment is 0.34 (electronic supplementary material, table S4). Given the contrasting strengths and weaknesses of the three experimental designs, it is not surprising that the overlap between candidate genes identified in each screen is limited. However, it is clear that the genetic architecture of incipient sexual isolation between Z and C *D. melanogaster* strains is highly polygenic.

We only used one Zimbabwe isofemale line (Z30) and the DGRP lines were derived from a single North America population. However, the original study reporting incipient sexual isolation between flies from Zimbabwe and cosmopolitan flies [3] used nine isofemale lines from Zimbabwe and eight C lines collected worldwide. All pairings of any Z female strain with any C male strain had much reduced mating success. Therefore, it is likely that our results are generalizable to other Z and C genotypes.

In addition to candidate variants and genes associated with mating success of DGRP males with Z30 females, we found that three common polymorphic inversions were associated with this trait. The association of *In(3R)K* with Z30 mating success is puzzling because it is the standard/inversion karyotype that is responsible for the association. Since polymorphic inversions in the DGRP are islands of genetic differentiation [13], we hypothesize that this association could be owing to heterozygote superiority for mating success of DGRP males in general, and not specifically associated with mating success with Z30 females. The associations of *In(2L)t* and *In(3R)Mo* are for the homozygous inversion karyotypes, and add to the growing literature on the role of inversions in speciation [34,35].

We functionally assessed the effects of knocking down gene expression of 17 candidate genes using *UAS*-RNAi constructs and three ubiquitous *GAL4* drivers with different strengths. RNAi of one candidate gene (*CG44837*) in C males resulted in increased mating to Z30 females, while RNAi of seven candidate genes (*C15*, *dpr1*, *CG1136*, *CG42672*, *btsz*, *jvl*, *nmo*) in C males resulted in decreased mating to Z30 females. Interestingly, *btsz* and *nmo* are also candidate genes for the amount of 5,9-heptacosadience in C females [21] (electronic supplementary material, table S9C). *btsz* (*bitesize*) encodes a synaptotagmin-like protein with annotated roles in actin filament organization, apical junction assembly, gastrulation (germ band assembly), morphogenesis of embryonic epithelium and lumen formation in the tracheal system [36,37]. *btsz* has not previously been annotated to affect behaviour, but it is expressed in the larval and adult central nervous system [38]. *nmo* (*nemo*) encodes a proline-directed serine/threonine kinase with multiple pleiotropic roles in the development, including eye [39] and wing [40] development and regulation of the Wnt signalling pathway [40]. *nmo* is expressed in adult brains [38] and affects gravitaxis behaviour [41]. *C15* encodes a transcription factor and affects antenna, chaeta and leg development [42,43]. *dpr1* (*defective proboscis extension response 1*) is involved in salt aversion, sensory perception of salty taste [44] and synapse organization [45] and is expressed in adult brains [38]. *jvl* (*javelin-like*) encodes a microtubule-associated protein which regulates mRNA localization during development, affects chaetae development [46] and is expressed ubiquitously, including the larval and adult central nervous system [38]. *CG1136*, *CG42672* and *CG44837* are computationally predicted genes for which there are no experimentally annotated functions, although all are expressed in the adult brain [38]. None of these candidate genes has been previously associated with mating behaviour, although they are plausible candidates based on brain gene expression.

Functional assessment of phenotypes using RNAi in *D. melanogaster* is facilitated by large numbers of publicly available *UAS*-RNAi stocks and a wide variety of *GAL4* drivers with different expression patterns [38]. However, RNAi cannot mimic the effects of candidate SNPs. For example, the effects of intronic SNPs in *mew* (*multiple edematous wings*) and an insertion/deletion polymorphism 47 bp upstream of the transcription start site of *Or67d* (*Odorant receptor 67d*) had among the largest effects and lowest *p*-values in the DGRP GWA analysis (electronic supplementary material, table S2). RNAi of *mew* resulted in lethality with the stronger *Ubi-GAL4* and *Act-GAL4* drivers that gave phenotypic effects for other genes. There was no effect of RNAi of *Or67d* with any driver, but it is a particularly interesting candidate gene because it is the receptor for the male-specific sex pheromone, 11-*cis*-vaccenyl acetate, which inhibits male and promotes female mating behaviour [47]. Rigorous functional assessment of the effects of the insertion/deletion polymorphism in *Or67d* or polymorphisms in other candidate genes affecting the mating behaviour of C males with Z females would entail either evaluating these associations using additional DGRP lines that were not used in this study [48] or creating scarless allelic replacements of the polymorphic alleles in a common homozygous genetic background. Our results raise the question of how candidate genes expressed in the nervous system along with those associated with sensory perception are functionally interconnected to mediate mating cues and how the genetic underpinnings of such functional ensembles enable the evolutionary trajectory that leads to incipient sexual isolation. The candidate gene and variant associations presented here provide testable hypotheses for future studies investigating the molecular genetic basis of incipient sexual isolation in *D. melanogaster*.

## Data Availability

Sequence data can be accessed through the SRA with accession numbers PRJNA1090936 and PRJNA1089210. Other data and codes are provided in the Dryad data repository [49] and the supplementary materials [50].
